# An overview of kinase downregulators and recent advances in discovery approaches

**DOI:** 10.1038/s41392-021-00826-7

**Published:** 2021-12-20

**Authors:** Beilei Wang, Hong Wu, Chen Hu, Haizhen Wang, Jing Liu, Wenchao Wang, Qingsong Liu

**Affiliations:** 1 Anhui Province Key Laboratory of Medical Physics and Technology, Institute of Health and Medical Technology, Hefei Institutes of Physical Science, Chinese Academy of Sciences, Hefei, 230031 People’s Republic of China; 2 Hefei Cancer Hospital, Chinese Academy of Sciences, Hefei, 230031 People’s Republic of China; 3 Hefei PreceDo pharmaceuticals Co., Ltd, Hefei, Anhui 230088 People’s Republic of China

**Keywords:** Drug development, Molecular medicine

## Abstract

Since the clinical approval of imatinib, the discovery of protein kinase downregulators entered a prosperous age. However, challenges still exist in the discovery of kinase downregulator drugs, such as the high failure rate during development, side effects, and drug-resistance problems. With the progress made through multidisciplinary efforts, an increasing number of new approaches have been applied to solve the above problems during the discovery process of kinase downregulators. In terms of in vitro and in vivo drug evaluation, progress was also made in cellular and animal model platforms for better and more clinically relevant drug assessment. Here, we review the advances in drug design strategies, drug property evaluation technologies, and efficacy evaluation models and technologies. Finally, we discuss the challenges and perspectives in the development of kinase downregulator drugs.

## Introduction

**Fig. 1:**
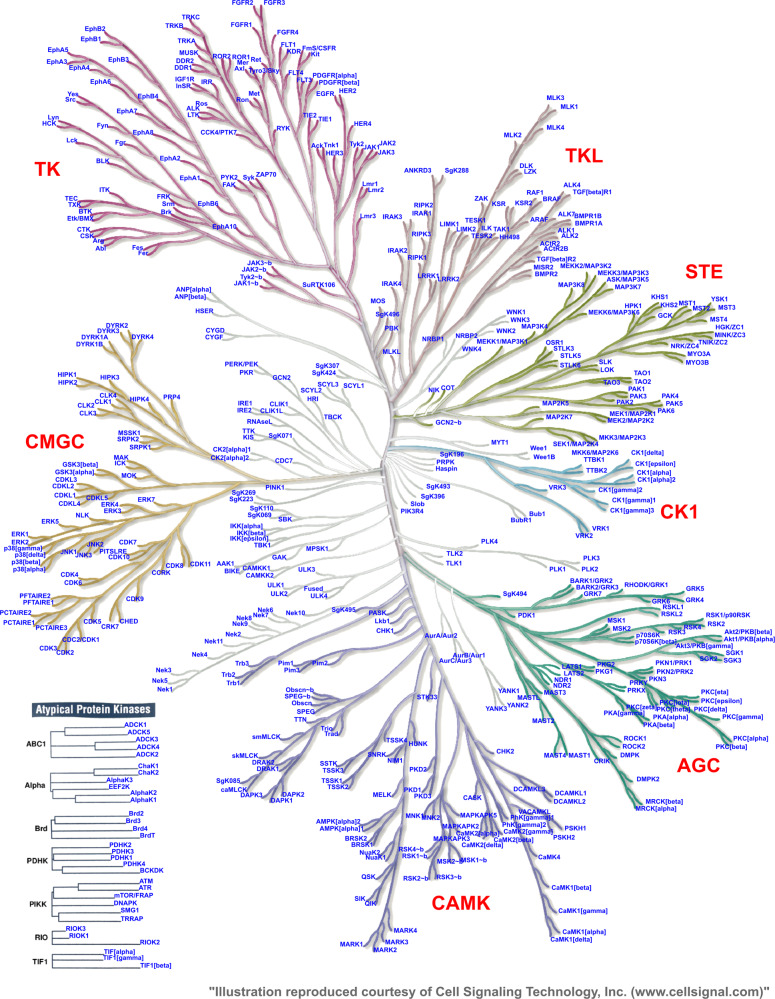
The KinMap of 518 human kinases. (The picture was obtained from http://www.kinhub.org/kinmap/index.html)

Kinases are crucial mediators of signal transduction processes, and by catalyzing the transfer of phosphates from ATP to other specific molecules, they are key regulators of a variety of cell functions. Protein kinases and lipid kinases constitute the human kinome. A total of 518 protein kinases containing 478 typical and 40 atypical kinases (Fig. 1) have been identified in the human genome.^1^ In general, kinases contain a eukaryotic protein kinase domain, and atypical kinases lack this protein kinase domain, but still exhibit kinase activity. Based on the sequence similarity of kinase domains, typical kinases are further classified into eight major groups: TK (tyrosine kinase, 90 members), TKL (tyrosine kinase-like kinase, 43 members), STE (STE7-, STE11-, and STE20-related kinase, 47 members), CK1 (casein kinase 1, 12 members), AGC (protein kinase A/G/C-related kinase, 63 members), CAMK (Ca^2+^/calmodulin-dependent kinase, 74 members), CMGC (CDK/MAPK/GSK/CDK-like-related, 61 members), and RGC (receptor guanylyl cyclase, 5 members). In addition, 40 atypical protein kinases have been identified, including pyruvate dehydrogenase kinases, bromodomain kinases, B cell receptors (BCRs), and PI3 kinase-related kinases.

The TK group consists of two families: receptor tyrosine kinases (RTKs) and non-RTKs.^2^ RTKs are transmembrane proteins that contain an extracellular segment and intracellular kinase domains. RTKs are activated upon dimerization, which is triggered by the binding of ligands to the extracellular segment; examples of RTKs include ErbB/HER family proteins, FLT3, PDGFRs, and FGFRs. Non-RTKs include SRC, ABL, and Janus kinase (JAK) kinases. Most members of the TKL family are serine/threonine kinases, with sequences similar to those of the TK group, and include IRAK, RAF, LIMK, and TGFβ.^3^ Three families of STE kinases have been identified on the basis of their homology with yeast proteins STE20 (MAP4K), STE11 (MAP3K), and STE7 (MAP2K, MEK), which regulate various signaling pathways.^4^ CK1 is also a Ser/Thr kinase associated with cytoskeletal function and transcription.^5^ In the human genome, AGC protein kinases are named after the protein kinase A, G, and C families (the PKA, PKC, and PKG families, respectively), which contain more than 60 protein kinases and are classified into 14 families; they regulate the signaling pathway of infectious diseases.^6^ Most members of the CAMK group are involved in calcium/calmodulin modulation, and they can be classified into four subfamilies: CaMK I, CaMK II, CaMK III, and CaMK IV.^7^ However, CHK1 and CHK2 are special classes of CAMK kinases, and both are cell cycle checkpoint kinases. CDKs, MAPK, GSK, and CDK-like kinases constitute the CMGC group, a diverse group of kinases. CDKs are involved in cell cycle progression and transcription. MAPKs play key roles in the proliferation, differentiation, death of cells, which contain three major groups: ERK, JNK, and p38. The functions of GSKs are rather different, which are involved in glycogen metabolism and inflammation.

**Fig. 2:**
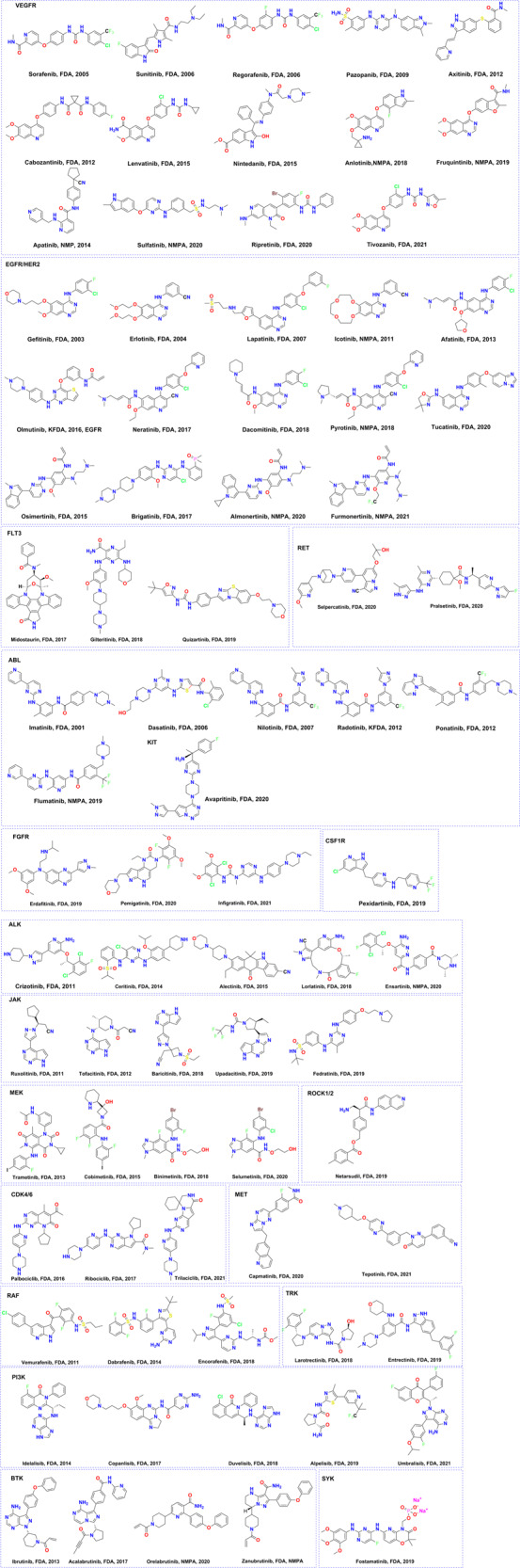
Chemical structures of approved kinase inhibitors

Kinase can transfer the phosphorylation of specific amino acids using ATP as the source of phosphate, the kinase domain contains special residues that promote the transfer of γ-phosphate from ATP to a protein or lipoprotein and the release of the products; this action requires a conformational change between the active (catalytically competent) and inactive states of the phosphate recipient. Approximately 250 amino acids in the N- and C-lobe constitute the kinase domain. One C-helix and several β-strands constitute the N-lobe; however, C-lobes mainly contain α-helixes that contain critical residues needed for kinase interaction with phosphate. A cleft, called a hinge, separates the N-lobe from the C-lobe, enabling ATP binding and phosphate transfer through residues that form hydrogen bonds with the adenine ring of ATP. The dysregulation of signaling pathways involving these kinases has been found in multiple diseases, e.g., cancer, inflammatory, degenerative, and infectious diseases.^8^ Tremendous efforts have been devoted to kinase-target drug discovery in the past three decades. As a result, many kinase inhibitors have been approved and are being used in clinical treatment, and others have been added to the rich pipeline of new molecules currently under clinical evaluation or preclinical development. Until August 2021, 3 natural macrocycles targeting mTOR and 68 other kinase inhibitors have been approved by the United States and Korea Food and Drug Administration (FDA),^9^ and 11 kinase inhibitor drugs have been approved by the National Medical Products Administration of China for pharmacological research, oncological applications, and inflammatory diseases (Fig. 2 and Table 1).

**Table 1:** Kinase inhibitors approved by FDA and NMPA

Year	Name	Binding mode	Target	PDB ID
2001	Imatinib	Type II	ABL/KIT/PDGFR	2HYY
2003	Gefitinib	Type I	EGFR	4WKQ
2004	Erlotinib	Type I	EGFR	4HJO
2005	Sorafenib	Type II	FLT3/KIT/RET/PDGFR/VEGFR	3WZE
2006	sunitinib	Type I	FLT3/KIT/RET/PDGFR	3G0E,6JOK
	Dasatinib	Type I	ABL/KIT/LCK/Src/PDGFR	3G6D
2007	Nilotinib	Type II	ABL/KIT/PDGFR	3CS9
	Lapatinib	Type1/2	EGFR/HER2	3BBT, 1XKK
2009	Pazopanib	Type I	KIT/VEGFR	PDB ID of its analog:^204^ 3CJG
2011	Icotinib	Type I	EGFR	
	Crizotinib	Type I	ALK/ROS/c-MET	3ZBF
	Ruxolitinib	Type I	Pan-JAK	Ref. ^205^
	Vemurafenib	Type I	BRAF	3OG7
2012	Axitinib	Type II	KIT/PDGFR/VEGFR	4AG8
	Radotinib	Type II	ABL	
	Bosutinib	Type I	ABL/Src	3UE4, 4MXO
	Regorafenib	Type II	FLT3/KIT/VEGFR/PDFGR	Ref. ^9^
	Tofacitinib	Type I	JAK	3LXK,3FUP
	Cabozantinib	Type II	FLT3/KIT/VEGFR/FGFR/PDFGR/RET/c-Met/TRK2	4MXC
	Ponatinib	Type II	ABL/FLT3/KIT/PDGFR/ FGFR/Src	3OXZ
2013	Afatinib	Covalent	EGFR/HER2	4G5J, 4G5P
	Ibrutinib	Covalent	BTK	6DI9
	Trametinib	Type III	MEK1/2	Ref. ^206^
2014	Ceritinib	Type I	ALK/ROS	4MKC
	Apatinib	Type I	VEGFR2	
	Idelalisib	Type I	PI3Kδ	4XE0
	Dabrafenib	Type1/2	BRAF-V600E	5CSW
2015	Alectinib	Type I	ALK	3AOX
	Cobimetinib	Type III	MEK1/2	4AN2
	Palbociclib	Type I	CDK4/6	5L2I
	Osimertinib	Covalent	EGFR	6JXT
	Lenvatinib	Type I	KIT/RET/FGFR/PDGFR/ VEGFR	3WZD, 5ZV2
	Nintedanib	Type I	KIT/FGFR/VEGFR	3C7Q
2016	Olmutinib	Covalent	EGFR	
2017	Brigatinib	Type I	ALK/EGFR/FLT3/IGF1R/ ROS1	BMX8
	Acalabrutinib	Covalent	BTK	Ref. ^207^
	Ribociclib	Type I	CDK4/6	5L2T
	Neratinib	Covalent	EGFR/HER2	2JIV, 3W2Q
	Midostaurin	Type I	FLT3/KIT	4NCT
	Copanlisib	Type I	PI3K	5G2N
2018	Anlotinib	Type I	FGFR/PDGFR/VEGFR	
	Lorlatinib	Type I	ALK	4UXL
	Fruquintinib	Type I	VEGFR	
	Binimetinib	Type III	BRAF	7M0U
	Pyrotinib	Covalent	HER1/2/4	Ref. ^208^
	Dacomitinib	Covalent	EGFR	4I24
	Gilteritinib	Type I	FLT3/ALX	6JQR
	Larotrectinib	Type I	TRK	Ref. ^209^
	Duvelisib	Unreported	PI3Kδ/γ	
	Fostamatinib	Type I	SYK	
	Encorafenib	Type I	BRAF/V600E	
	Baricitinib	Unreported	JAK1/2	
2019	Pexidartinib	Type II	CSF1R/KIT/FLT3	4R7H
	Zanubrutinib	Covalent	BTK	6J6M
	Entrectinib	Type I	TRK	5FTO
	Erdafitinib	Type I	FGFR	5EW8
	Quizartinib	Type II	FLT3	4XUF
	Fedratinib	Type I	JAK2	6VNE
	Alpelisib	Type I	PI3Kα	4JPS
	Upadacitinib	Unreported	JAK1	
	Flumatinib	Type II	ABL/KIT/PDGFR	
	Netarsudil	Unreported	ROCK1/2	
2020	Pemigatinib	Type I	pan-FGFRs	
	Avapritinib	Unreported	KIT/PDGFR	
	Ripretinib	Type II	KIT/PDGFR	6MOB
	Selumetinib	Type III	MEK1/2	4U7Z
	Capmatinib	Unreported	MET	
	Tucatinib	Unreported	HER2	
	Almonertinib	Covalent	EGFR	Ref. ^208^
	Ensartinib	Unreported	ALK	
	Orelabrutinib	Covalent	BTK	Ref. ^210^
	Sulfatinib	Unreported	VEGFR/FGFR	
	Selpercatinib	Type I	RET	7JU6
	Pralsetinib	Type I	RET	7JU5
2021	Trilaciclib	Unreported	CDK4/6	
	Furmonertinib	Covalent	EGFR	Ref. ^211^
	Infigratinib	Type I	FGFRs	3TT0
	Tepotinib	Type I	MET	4R1V
	Tivozanib	Type II	VEGFR2	4ASE
	Umbralisib	Type I	PI3Kδ	

Generally, kinase inhibitors can be categorized into two classes according to whether ATP competition or non-ATP competition or not.^3,10^ The potencies of kinases that compete for ATP decrease with increasing ATP concentration, and these kinases can be classified as type I and type II inhibitors.^3,11^ Type I inhibitors bind to the active (DFG-in) conformation, whereas type II inhibitors bind to the inactive (DFG-out) conformation. The potency of non-ATP-competitive kinases is unchanged at different ATP concentrations, and they are also called allosteric inhibitors. Type I inhibitors bind to the ATP-binding pocket through the formation of hydrogen bonds to the kinase “hinge” residues, which are just like the interaction of adenine with the kinase. In addition, hydrophobic interactions in and around the region occupied by the adenine ring of ATP are also important. However, type II inhibitors not only occupy the ATP-binding pocket but also exploit unique hydrogen bonds with the residues of α-helix and DFG motif. In addition, the special hydrophobic interactions formed by the DFG motif of the activation loop being folded away from the conformation required for ATP phosphate transfer can improve the selectivity among the kinases. In most cases, the number of hydrogen bonds between kinases and inhibitors in type II inhibitors is more than that of type I inhibitors. Typically, type I inhibitors have a heterocyclic motif that forms 1–3 hydrogen bonds at the hinge area. Similar to type I inhibitors, the hinge binding part needs heterocycle to form the typical hydrogen bonds with target kinase. Type II inhibitors can be divided into five parts from the chemical structure, e.g., head, hinge binding, linker, hydrogen bond, and tail. The moiety of the head can be hydrophobic or hydrophilic. The linker part needs a hydrogen donor or acceptor, e.g., amido linkage, urea bridge bonds, and so on, which can form special hydrogen bonds with the residues of α-helix and DFG motif. The final “tail” segment contains a hydrophobic moiety that occupies the pocket created by the DFG-out flip. Allosteric inhibitors are described in more detail in the following section. By analyzing the binding modes of small molecules to kinases, we found that most inhibitors adopt type I binding modes, only four MEK inhibitors adopt type III binding modes (Table 1). From the co-crystallization data published on PDB, 29 co-crystal complexes of small molecules and kinases employ type I binding modes, 11 adopt type II binding modes. In addition, nine small molecules are reported as irreversible inhibitors by forming covalent bonds with kinases (Table 1), which will be discussed in more detail in the following sections.

In spite of the booming development of kinase inhibitors, issues with available drugs still exist, such as the high failure rate during development, side effects, and drug-resistance problems. To cope with these, multidisciplinary technologies and new approaches have been applied to solve the above problems during the discovery process of kinase inhibitors. In this review, we summarize the advances from three directions, including advances in drug design strategies, drug property evaluation technologies, and efficacy evaluation models and technologies. Finally, we discuss the challenges and perspectives of the future development of kinase inhibitors.

## Advances of kinase inhibitors design strategies

Acquired drug-resistance problems driven by evolution pressure during small-molecule kinase inhibitor treatment become a major unmet clinical need in cancer therapy. Almost all the protein kinases drugs will face drug resistance after a period of time of clinical use. To address the problem of drug resistance, many strategies have been developed, e.g., forming a covalent bond to improve the affinity, degradation mutant kinase by new approaches, and overriding ATP-binding site mutations with allosteric inhibitors.

### Covalent binding approach

**Fig. 3:**
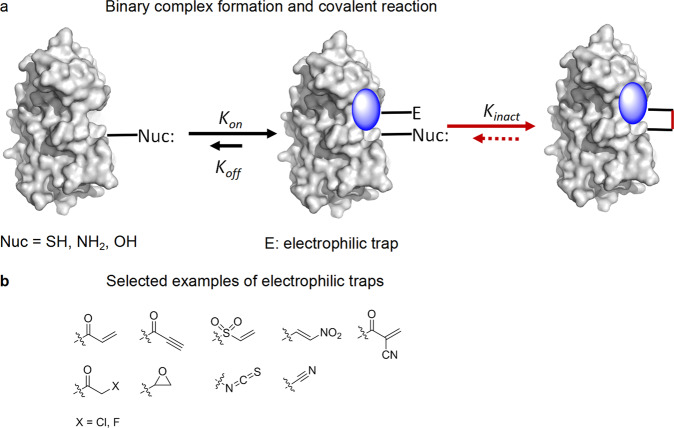
Mechanisms of irreversible covalent inhibitors and the selected examples of electrophilic traps. **a** Small molecules form a covalent bond with the specific nucleophilic residue of the target protein kinase via reaction with an electrophile; then, the equilibrium will be interrupted and tend to complex formation with the inhibitor after the conjugation through an irreversible covalent bond. **b** Selected widely used examples of electrophilic traps are shown

Traditionally, compared with the traditional reversible binding inhibitors, the induction of covalently binding drugs can reduce inevitable side effects due to possible off-target and improve the specific activity, and covalently binding drugs have been widely used; compound forms a covalent bond with a specific nucleophilic residue on the target protein kinase via reaction with an electrophile. Initially, the interaction of small molecules and kinase is in a dynamic equilibrium state. The equilibrium will be interrupted and tend to complex formation with inhibitor after the conjugation through an irreversible covalent bond (Fig. 3). In addition, the higher (or infinite) binding affinity obtained by covalently binding drugs is known to lead to longer residence time and induce protein degradation, which can completely block signaling pathways. Particularly after the early success of the epidermal growth factor receptor (EGFR) inhibitor afatinib, increasing efforts have been directed to the covalent binding approach in the past decade. The most critical point for designing covalent kinase inhibitors is to find suitable targetable amino acids in the binding pocket. In most cases, covalent binding kinase inhibitors are designed to form chemical bonds with the appropriate cysteine thiols via Michael addition reactions.^12,13^ To date, eight covalent inhibitors have been approved by the FDA (Fig. 2), all of which are designed to target cysteine thiols. Recently, considerable efforts have been made to explore alternative electrophiles that exhibit irreversible or reversibly covalent binding mechanisms toward other amino acids, such as lysine,^14^ aspartic acid,^15^ and others.^16^

**Fig. 4:**
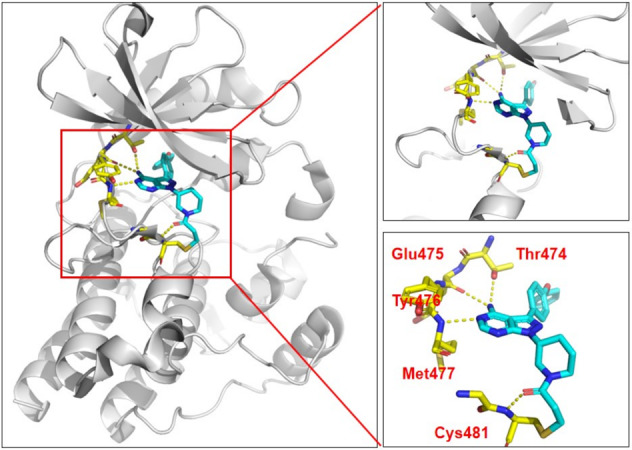
The binding mode of ibrutinib with BTK (PDB: 5P9J). The N atom and NH_2_ of the purine ring form three hydrogen bonds with Glu475, Tyr476, and Thr474, respectively, to fix ibrutinib. The carbonyl forms a hydrogen bond with Cys481, at the same time the allyl and Cys481 forms a covalent bond via Michael addition

Nearly half of eukaryotic kinases contain at least one cysteine residue neighboring the ATP pocket, e.g., EGFR and Bruton tyrosine kinase (BTK). To overcome the first-generation drug resistance caused by gatekeeper T790M mutation, the second-generation EGFR inhibitors (e.g., afatinib,^17^ dacomitinib,^18^ neratinib^19^) and the third-generation EGFR inhibitors (e.g., osimertinib^20^) were designed, which can irreversibly bind to EGFR C797 residue and each contains an α, β-unsaturated acrylamide electrophilic warhead. However, new point mutation C797S appeared, which disrupted the formation of covalent bonds; thus, new scaffold drugs are needed. In 2013, the first approved BTK inhibitor ibrutinib has a classical covalent binding mode, and binds to BTK in the ATP pocket and forms one covalent bond with Cys481 and four hydrogen bonds with Thr474, Glu475, Tyr476, Met477, and Cys481, respectively (Fig. 4). In 2017, the second BTK inhibitor acalabrutinib was approved. Both BTK inhibitors form a covalent bond with Cys481 residue in the BTK activation site through an acrylamide electrophile warhead.^21,22^ Acalabrutinib possesses a unique and relatively inert 2-butynamide electrophile, which is thought to be activated by its relative positioning to the electron-withdrawing imidazopyrazine core. However, it should be noted that 11 kinases including EGFR, ITK, BTK, JAK3, and others share an equivalently positioned cysteine residue that can be targeted through the covalent binding approach, which makes it hard to get high selectivity between them.

Some efforts have been made to target active site lysine residues in certain kinases in which cysteine residues are unsuitable for covalent binding. Lysine is an attractive nucleophile because of its prevalence in many active sites and at interfaces mediating protein–protein interactions.^23–25^ Several studies have reported the covalent modification of lysine residues using various warheads. A sulfonyl fluoride-based probe was recently used to covalently combine with Lys514 of FGFR.^25^ Ester electrophiles were recently reported to form a covalent bond with Lys779 in the P-loop of PI3Kδ.^26^ Other amino acids, such as tyrosine and aspartic acid, can also serve as potential nucleophilic agents depending on the microenvironment. The first irreversible serine–arginine protein kinase (SRPK1) inhibitor targeted a tyrosine residue with a sulfonyl fluoride warhead.^27^ An EGFR inhibitor can form a unique covalent interaction with the boronic acid moiety through Asp800 at the EGFR kinase hinge region.^28^ However, rapid selection pressure inside the cells makes the drug-resistant mutation occur, e.g., patients treated with irreversible kinase inhibitors such as ibrutinib and osimertinib usually develop resistance due to cysteine residue mutation.^29,30^ Therefore, people have to design new drugs through new strategies to solve the drug-resistance issues.

### Protein degradation approach

**Fig. 5:**
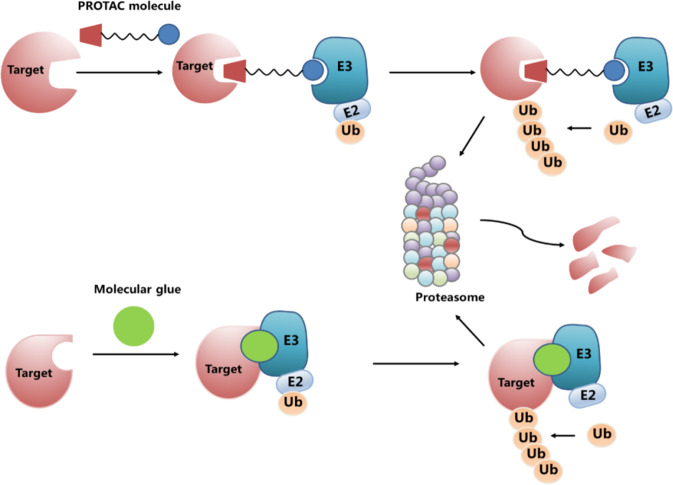
Schematic diagram of targeted protein degradation strategies. PROTACs are heterobifunctional small molecules consisting of a ligand for the target protein and another ligand for the E3 ubiquitin ligase, which are connected via a linker. Molecular glue is a class of small-molecule drugs that promotes the interaction between disease-causing target proteins and ubiquitin ligase complexes and marks the targeted protein for degradation

To show the important role of catalytic kinases in the signal transduction pathway, reports have indicated that kinases play roles in cancers independent of their catalytic function. Notably, traditional small-molecule inhibitors that do not modulate the noncatalytic function of kinases cannot block the proliferation of tumor cells that rely on the noncanonical role of kinases. Promotion of kinase degradation can lead to the total loss of target function, including both catalytic and noncatalytic functions. Thus, using small-molecule kinase inhibitors to downregulate the protein levels of the kinase is an emerging therapeutic modality in cancer therapy. The ubiquitin–proteasome system is one of the main pathways critical for protein degradation. Molecular glue degraders and proteolysis targeting chimeras (PROTACs) (Fig. 5) are the main targeted protein degradation tools used to induce ubiquitin-dependent degradation of proteins through small molecules.

PROTACs constitute a promising technology for the selective degradation of cellular proteins. PROTACs are heterobifunctional small molecules consisting of a ligand for the target protein and another ligand for the E3 ubiquitin ligase, which are connected via a linker.^31^ PROTACs recruit the E3 ligase to the protein of interest and trigger ubiquitination and subsequent degradation of the target by the ubiquitin–proteasome system. With the successful development of small-molecule E3 ligands, many ligands for MDM2, VHL, CRBN, and cIAP have been used as the basis for PROTAC development against various cancer targets.^32^ Since the concept of PROTACs was raised by Deshaies’s team in 2001,^33^ more than 60 proteins in different protein families and structure classes have been successfully degraded by PROTACs, including transcription-related kinases CDK8 and CDK9,^34,35^ proto-oncogene kinases BCR-ABL^36^ and FLT3,^37^ the inflammation-related kinase PIPK2,^38^ and the immunity-related kinase TBK1.^39^ The breakthrough in PROTAC technology led to two orally bioavailable PROTACs (ARV-471 and ARV-110), which have been evaluated in phase 1/2 clinical trials in patients with locally advanced or metastatic ER+/HER2− breast cancer and metastatic castration-resistant prostate cancer (NCT04072952 and NCT03888612); the early data on safety, tolerability, and efficacy are encouraging.

Recently, as a promising new drug design technology, PROTAC attracts more and more attention. PROTACs present several advantages, including induction of isoform-selective degradation, elimination of both enzymatic and scaffolding functions, ability to target members of multicomponent complexes for degradation, and conversion of promiscuous ligands into selective degraders.^40^ PROTACs have also been proven to overcome drug resistance by degrading targets. BTK plays a crucial role in the BCR signaling pathway and has been validated as a major drug target for B cell-related malignancies.^41^ Ibrutinib was the first covalent BTK kinase inhibitor approved by the FDA, and it has been approved for the treatment of mantle cell lymphoma (MCL), Waldenström’s macroglobulinemia, chronic lymphocytic leukemia (CLL), etc^21^ However, BTK C481S drug-resistant mutations have been found in several MCL or CLL patients after long-term treatment of ibrutinib.^42^ BTK PROTAC P13I consists of ibrutinib with pomalidomide to recruit CRBN, which can effectively degrade wild-type and mutant BTK C481S. Furthermore, P13I efficiently inhibited the proliferation of ibrutinib-resistant BTK C481S and BTK WT DLBCL cell lines. The results of this study suggest that PROTAC may be a promising strategy for treating mutation-induced drug resistance.^43^

Nevertheless, various challenges have limited PROTAC applications, including difficulties in rational design, metabolic instability, poor cellular permeability, off-target toxicity, and large molecule weights. Researchers have adopted several creative strategies to overcome these barriers. Many efforts have been made to optimize the pharmacokinetic (PK) and pharmacodynamic properties of PROTAC. Since the length, rigidity, and hydrophilicity of the linker play important roles in determining the property of PROTAC. A systematic medicinal chemistry campaign took place to improve the PK properties of BTK PROTAC by optimizing the linker. The results show that the presence of ether moiety in the linker improved the PK characteristics and show good antitumor activity in vivo.^44^ It is also reported that using triazole as a linker can improve the metabolic stability and antitumor activity in vivo;^45^ “click chemistry” is used for the rapid synthesis of molecules to induce protein degradation. Through the rapid chemical reaction in cells, two molecules are paired to form a PROTAC. Both of these proteins have lower molecular weights and better cell permeability than the original PROTAC. For a proof-of-concept for this “click chemistry” platform, the JQ-1-IMiD PROTAC was synthesized to successfully degrade the BET bromodomain.^46^ Besides, many efforts have been made to improve the selectivity of PROTAC. Distinct E3 ligases may have different lysine selectivity profiles, which alter the selectivity of PROTAC with different E3-recruiting moieties as well. Lai et al.^47^ reported that PROTACs recruiting CRBN can degrade both BCR/ABL and c-Abl, whereas c-Abl can be only degraded by VHL-recruiting PROTAC. Also, a stable ternary complex is another key to achieving the selectivity of PROTACs. c-Abl can be effectively degraded by PROTAC with appropriate linker length between VHL and c-Abl, but longer linkers may reduce degradation efficiency due to the unfavored protein–protein interaction between VHL and c-Abl. To obtain a deeper understanding of the key molecular events in PROTAC discovery and optimization, an experimental map of degradable kinases in all clades of the kinome and chemical leads for more than 200 distinct kinases has been established and presented as a public resource (https://proteomics.fischerlab.org). The large dataset shows that ligase binding moiety, ternary complex formation, linker length, cellular target occupancy, and target expression are important factors that should be considered in PROTAC development. This work provides guidance for predicting proteins that can be degraded and a rational design method for more effective discovery of active degraders.^48^ In addition, most of the reported PROTACs are based on noncovalent binding to the target protein. Covalent binding can enhance the binding affinity to the targeted protein. Several studies have used covalent ligands against target proteins in the PROTAC design, such as BLK, KRAS^G12C^, and BTK, these targets were successfully degraded by covalent PROTACs.^49–51^ Recently, reversible noncovalent, reversible covalent, and irreversible covalent BTK inhibitors are compared side by side to see how different warheads affect degradation efficiency. The results showed that reversible covalent ligands can significantly improve intracellular accumulation and target binding, and the reversible binding maintains the catalytic properties of PROTACs, making them preferable to irreversible ligands.^52^

Molecular glue is a class of small-molecule drugs that promotes the interaction between disease-causing target proteins and ubiquitin ligase complexes and marks the targeted protein for degradation. Thalidomide analogs and arylsulfonamides are two classes of drugs that act in molecular glue degradation. Thalidomide is used for the treatment of multiple myeloma (MM). It was discovered that thalidomide works by repurposing the CRL4 CRBN ubiquitin ligase to degrade transcription factors IKZF1/3.^53,54^ Furthermore, kinase casein kinase Iα can be degraded by thalidomide through the CRL4^CRBN^ ubiquitin ligase.^55^ Thus, thalidomide and thalidomide analogs can be used to treat myelodysplastic syndrome with a deletion in chromosome 5q in the clinic. The mechanistic findings for thalidomide provided clinical proof-of-concept for targeted protein degradation therapeutics. Indisulam is an arylsulfonamide with anticancer activity. Similar to thalidomide, the mechanism of action is to recruit RBM39 (RNA-binding motif protein 39) to the CRL4^DCAF15^ E3 ubiquitin ligase, resulting in RBM39 polyubiquitination and proteasome degradation. These degraders work by exploiting the complementary protein–protein interfaces between the receptor and target.

Compared to PRAOTAC, molecular glue has a lower molecular weight, higher cell permeability, and better oral absorption, which makes it more drug-like.^56^ Although molecular glue degradation agents are very desirable, they are discovered mostly by chance, and the strategies used to identify or design these compounds are limited. Recently, it was found that the CDK inhibitor CR8 can be used as a molecular glue to depredate cyclin K. The compound has a solvent-exposed pyridyl moiety, which enables the formation of a complex between CDK12–cyclin K and the CUL4 adaptor protein DDB1, inducing ubiquitination and degradation of cyclin K.^57^ This study shows that modifying the surface exposure region where small molecules bind to the target is a reasonable strategy that can be used to develop molecular glue degradation agents for protein targets of interest.^58^ Also, a rational discovery of molecular glue degraders via scalable chemical profiling has been reported. The authors presented differential chemical profiling in hypomethylated cell lines with a promiscuous deficiency in CRL activity. Using this method, they successfully discovered a new molecular glue degradation agent that can induce cyclin K degradation through the CRL4B ligase complex. This research provides a rational strategy for identifying new cellular active molecular glue with a known mechanism.^59^

### Allosteric binding approach

The majority of approved and preclinical kinase inhibitors are type I or type II compounds that bind to the ATP pockets. However, due to the high sequences and structural similarities among ATP pockets of human kinases, it is difficult for inhibitors to achieve high selectivity, and poor selectivity usually causes side effects. In addition, acquired drug resistance such as mutations of amino acids blocks the drugs from binding to target kinases. Allosteric kinase inhibitors with targets outside the highly conserved ATP pocket have been proposed as promising tools to overcome barriers to using kinase inhibitors, including poor selectivity and drug resistance. Since the first small-molecule allosteric inhibitor trametinib was approved in 2013, a number of clinical trials of more than ten allosteric inhibitors are ongoing, and an increasing number of highly selective and potent preclinical allosteric kinase inhibitors are being developed.

**Fig. 6:**
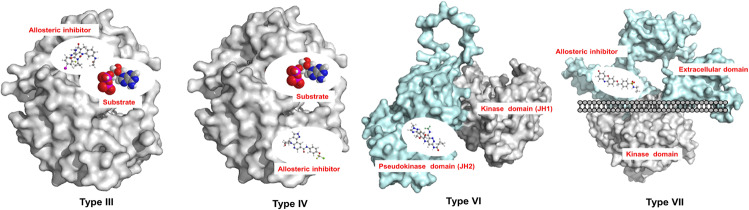
The four types of allosteric kinase inhibitors. Allosteric inhibitors bind to allosteric pockets that are adjacent to the ATP pocket, but do not overlap with the ATP-binding pocket, which is defined as type III inhibitors. The binding pockets are distant from the ATP-binding pocket; these kinds of small molecules are defined as type IV inhibitors. Small molecules that bind the pseudokinase domain (JH2) are defined as type VI inhibitors and these bind the extracellular domain (ECD), which are defined as type VII

**Fig. 7:**
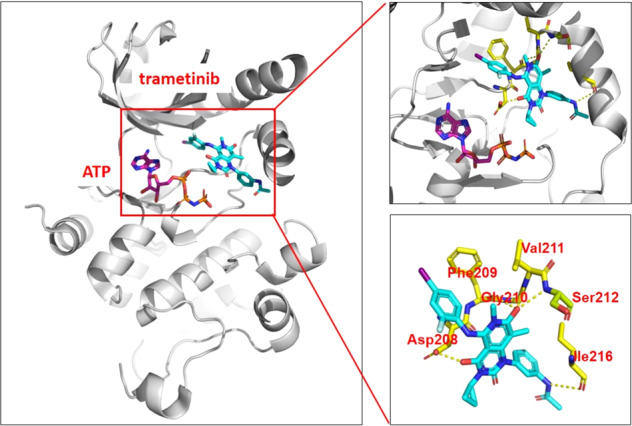
The binding mode of trametinib with MEK1 (PDB: 7M0Y). Trametinib binds with MEK1 in type III binding mode at the allosteric pocket next to the ATP pocket and forms five hydrogen bonds with Asp208, Phe209, Gly210, Val211, Ser212, and Ile216

Allosteric inhibitors bind to allosteric pockets that are adjacent to the ATP pocket, but do not overlap with the ATP-binding pocket, also defined as type III inhibitors, or these are distant from the ATP-binding pocket, also defined as type IV inhibitors (Fig. 6). Three type III allosteric MEK1/2 inhibitors, trametinib,^60^ cobimetinib,^61^ and binimetinib,^62^ have been approved for use as single agents or in combinations with Raf inhibitors for the treatment of solid tumors patients, and all bind to the allosteric MT3 site of the kinase domain encompassed by the Lys97, Ser212, and Val127 residues of MEK1.^63,64^ For example, trametinib fits well with MEK1 in type III binding mode at the allosteric pocket next to the ATP pocket and forms five hydrogen bonds with Asp208, Phe209, Gly210, Val211, Ser212, and Ile216 (Fig. 7). Approximately 20% of patients treated with single-agent trametinib showed specific side effects, such as rash, diarrhea, and lymphedema. To overcome these side effects, the combination of trametinib with the BRAF inhibitor dabrafenib was approved in 2014, but this combination did not decrease the overall toxic effects of trametinib treatment.^65^ Cobimetinib is a diarylamine compound that led to improved median progression-free survival. Selumetinib has approved in the United States for pediatric patients with neurofibromatosis type 1 plexiform neurofibromas in 2020; however, it failed to show positive therapeutic effects in a phase III trial for the treatment of KRAS mutant lung cancer. In addition, some other allosteric inhibitors with structures similar to that of cobimetinib and binimetinib have been used for the treatment of solid tumors at different stages of clinical evaluation, e.g., selumetinib, pimasertib, and PD0325901. More than ten allosteric small-molecule MEK inhibitors have entered clinical trials.^66^ Although these type III MEK inhibitors have markedly improved patient survival, allosteric mutations eventually occur, which dramatically reduced binding affinity. A recent study revealed that the allosteric pocket “gatekeeper’ MEK1 V211D mutation situated within the arylamine-binding pocket disrupts the binding of multiple allosteric inhibitors and eventually leads to drug resistance.^67^ Allosteric EGFR-T790M inhibitors such as EAI001 is a new type of EGFR inhibitor that binds to EGFR kinase adjacent to the ATP-binding site. The crystal structure of the inhibitor–EGFR complex shows that the compound binds an allosteric site created by the displacement of the regulatory C-helix in the inactive conformation of the kinase.^68^ EAI001 inhibits mutant EGFR L858R/T790M—with low-nanomolar potency in biochemical assays, but as a single agent, it is not effective in blocking EGFR-driven proliferation of cells. In addition, JBJ-04-125-02^69^ also binds to the allosteric pocket adjacent to the ATP-binding pocket. Some allosteric AKT inhibitors, such as MK2206,^70^ ARQ902,^71^ and borussertib,^72^ binds to the interface of the allosteric pocket of AKT formed by the kinase domain and the PH domain. The complex of borussertib and AKT irreversibly stabilize the inactive PH conformation. Borussertib is also a first-in-class covalent-allosteric AKT inhibitor that is used to treat small cell lung cancer in combination with topotecan in the clinic (NCT04768296). The ABL inhibitors GNF-2^73^ and asciminib (ABL001)^74^ bind to an allosteric pocket distant from the ATP-binding pocket, both are defined as type IV inhibitors. Asciminib (ABL001) was the first allosteric BCR-ABL inhibitor to enter the clinic. Asciminib binds to the myristate pocket of BCR-ABL and maintains activity against RTK inhibitor (TKI)-resistant ATP-site mutations.

JAK family members contain a distinctive catalytically inactive pseudokinase domain (JH2) that is similar in structure to the JH1 domain and includes an ATP-binding site, but it lacks catalytic activity.^75^ JAK family proteins play important “auto-inhibitory” roles in regulating the activation of the JH1 kinase domain, which makes this allosteric site an ideal target for the design of selective JAK inhibitors, and this type of binding mode was regarded as type VI. AT9283, a multitarget kinase inhibitor with potent aurora kinase activity, binds the pseudokinase-kinase domain of JAK2.^76^ Among JAK family members, BMS-986165 has been reported to be a selective allosteric TYK2 inhibitor that exhibits high selectivity, shows excellent PK properties with minimal profiling liabilities, and is efficacious in several murine models of autoimmune disease in clinical trials.^77^ SSR128129E was the first reported small molecule known to bind the extracellular domain (ECD) of FGFR, and crystallography combined with flexible docking and metadynamics studies demonstrated that SSR128129E binds the nonflexible H2 state of D3 in the ECD of FGFR2,^78^ which was defined as type VII. In addition, a highly selective DDR2 inhibitor that also targets the ECD of DDR2 inhibits DDR2–collagen binding interactions via allosteric modulation of the kinase.^79^

Overall, allosteric kinase inhibitors have several significant advantages over traditional orthostatic ATP-binding inhibitors, including improved activity, greater selectivity, lower off-target toxicity, and the ability to overcome the resistance mutations of ATP-binding pocket. Despite the advantages, challenges still exist. For example, as mentioned above, accumulating evidence shows that drug-resistance mutations also occur at the allosteric sites. In addition, the main reason that limits the discovery of allosteric inhibitors may be the finding of allosteric sites and the difficult rational design of allosteric inhibitors. Computational-aided approaches are helpful to address the emerging problems such as the identification of allosteric sites by AlloDriver^80^ and SBSMMA (structure-based statistical mechanical model of allostery).^81^ More importantly, a comprehensive understanding of allosteric-resistant mechanisms is crucial so that we can solve the emerging drug-resistance problems with allosteric inhibitors and guide future endeavors to overcome such barriers.

### Macrocycles design approaches

**Fig. 8:**
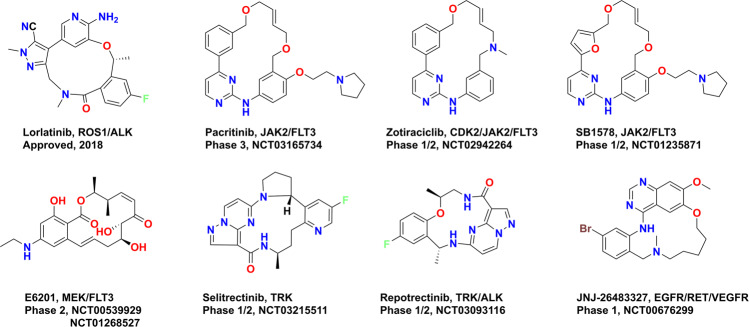
Macrocyclic kinase inhibitors approved and in clinical trials

In the past few years, macrocycles have attracted increasing attention in the discovery of kinase inhibitors. The macrocycles not only offer an opportunity for chemical novelty than the existing scaffolds but also improve the selectivity and overcome the acquired drug resistance.^82^ So far, except for the three mTOR inhibitors, only one ALK/ROS1 dual kinase inhibitor lorlatinib has been developed via rational design, which is a third-generation ALK/ROS1 inhibitor and it can overcome gatekeeper and solvent-front mutations.^83^ To avoid steric hindrance with a gatekeeper and solvent-front mutations, lorlatinib is designed using a rigid amide-linked 12-membered macrocycle based on the structure of crizotinib. The selectivity and activity were also improved at the same time. It is worth noting that lorlatinib has good blood–brain barrier penetration and central nerve system exposure, these properties lead to its applications as an effective agent against metastatic brain cancer.^84^ Currently, many macrocycles kinase inhibitors entered clinical trials (Fig. 8). Pacritinib, zotiraciclib, and SB1578 are all 2-aminopyrimidine-based macrocycles targeting JAK2/FLT3 kinases. But their clinical applications are different; pacritinib is being evaluated for the treatment of myelofibrosis and lymphoma in phase III^85^ and zotiraciclib is used for leukemia and MM.^86^ Different from pacritinib and zotiraciclib, the clinical applications of SB1578 are autoimmune diseases and inflammatory disorders such as psoriasis and rheumatoid arthritis.^87^ The resorcyclic lactone E6201 is a MEK and FLT3 dual inhibitor^88^ that entered clinical trial in 2007 for the treatment of plaque psoriasis,^89^ and then the other application in malignant melanoma was initiated in 2017. To overcome the mutations of TRK, two macrocyclic compounds were designed using macrocycle strategy based on the structure of larotrectinib.^90,91^ Both selitrectinib and recrotrectinib contain a pyrazolopyrimidine structure and entered clinical trials in 2017 for the treatment of advanced solid tumors. Recrotrectinib, a next-generation ROS1, pan-TRK, and ALK kinase inhibitor, overcomes resistance due to acquired solvent-front mutations involving ROS1, NTRK, and ALK. The macrocycle was introduced to increase conformation rigidity based on the larotrectinib scaffold, and it was used in patients with advanced solid tumors harboring ALK, ROS1, or NTRK1-3 rearrangements.^90,91^ JNJ-26483327 contains a quinazoline scaffold and entered phase I clinical trial in 2008 as a multitargeted tyrosine kinase inhibitor for the treatment of advanced and/or refractory solid malignancies.^92^ Although macrocyclic kinase inhibitors offer many advantages such as offering chemical novelty, the ability to overcome drug resistance, and improved selectivity, the challenges in chemical synthesis slow down its structure–activity relationship (SAR) optimization process.

## Advances of property evaluation technologies

Kinase family members share a conserved ATP-binding domain and exhibit high structural similarity, rendering it a great challenge to find selective kinase inhibitors. On account of the function of kinases in normal cells, usually multitarget inhibitors induce side effects due to off-targets. Kinomics selectivity analysis of kinase inhibitors is important for better understanding of inhibitor biological activity, obviating off-target activities, and, in some cases, identifying new targets that may lead to novel therapeutic applications. In the following section, we will review the profiling technologies in three categories including probe-based, probe-free, and catalytic activity-based.

### Probe-based selective kinome profiling technology

**Fig. 9:**
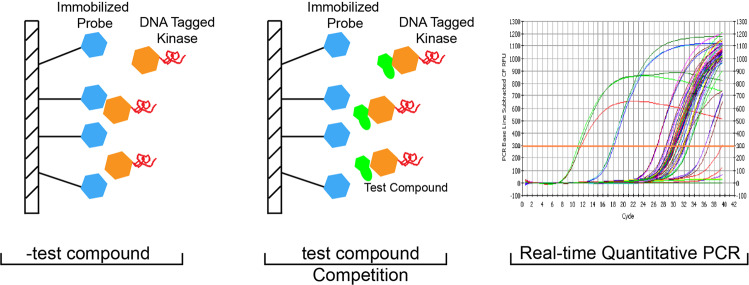
Schematic overview of competition binding assay. In this assay, DNA-tagged kinases are incubated with immobilized probes that bind kinases at the ATP-binding pocket. Then, a free test compound is introduced to compete with the probe to bind its target kinase, and the selectivity of a test compound is evaluated by quantifying the DNA-tagged kinase associated with the solid bead by real-time quantitative PCR

To evaluate the selectivity and activity of compounds against multiple kinases in a single experiment, a competitive binding assay based on phage displays and chemical probes was developed.^93^ In this assay, a probe that binds to multiple kinases at the ATP-binding pocket is immobilized on solid beads and incubated with DNA-tagged kinases. Then, a free test compound is introduced to compete with the probe to bind its target kinase, and the selectivity of the test compound is evaluated by quantifying the DNA-tagged kinase associated with the solid bead by real-time quantitative PCR (Fig. 9). Commercial profiling services, such as KINOMEscan, which can test most kinases (with coverage of more than 80% of the “typical” human protein kinome), are available.^94^ Using this approach, 10 μM 72 kinase inhibitors have been screened against 113 kinases and profiled, with the quantitative binding constant subsequently determining their targets. The compounds were quantified by measuring a selectivity score (*S* score). The results showed that, compared to type I inhibitors, type II inhibitors exhibited better selectivity.^94^ This analysis facilitated structural optimization and accelerated the drug development process. However, as it was performed in vitro without ATP or cofactors, the affinity as determined might not be correlated with that in cells.^95,96^ Loss of activity can be attributed to the protein structure, complex formation in the native environment, low cell permeability, or compound efflux.

The utilization of native proteins in profiling can recapitulate the physiological binding mode most accurately. Several chemical proteomic platforms have been developed for use in direct profiling of endogenous kinases in cellular and tissue proteomes via liquid chromatography-tandem mass spectrometry (LC-MS/MS).

**Fig. 10:**
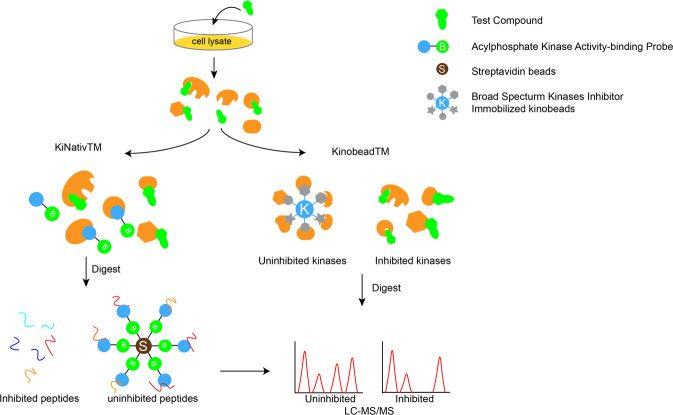
Chemical proteomic strategies for kinase inhibitor’s selectivity analysis in native proteomes. In these technologies, the test compound is preincubated with cell lysate, and then probes are introduced to enrich the uninhibited kinases. For KiNativ^TM^ approach (left), biotinylated acyl phosphate probes that bind kinases covalently are utilized; in Kinobead^TM^ (right), uninhibited kinases are precipitated with broad-spectrum kinases inhibitors immobilized on Kinobeads, and then enriched proteins or peptides are analyzed by LC-MS/MS

KiNativ is an in situ profiling platform based on a biotinylated ATP acyl phosphate probe, which covalently reacts with conserved lysine residues in the ATP-binding pocket of kinases.^97,98^ Test compounds are preincubated with a cell lysate, then probes are introduced to enrich the uninhibited kinases. The test inhibitor competes with the probe, and then uninhibited kinase proteins in cell lysates covalently bound with the probe are enriched by streptavidin and quantitated by LC-MS/MS (Fig. 10, left).^2,99^ Profiling by KiNativ demonstrated that CDKN2A-modulated CDK4 prevents target engagement of palbociclib,^100^ which correlated with the phenotype in which a high level of CDKN2A is associated with reduced sensitivity to palbociclib.^100,101^ This result indicated that profiling of CDK kinases in cell lysates is more accurate than in vitro biochemical assays, as CDKs are associated with cofactors that might influence the interaction of compounds and kinases.

Kinobeads are used in an approach similar to that of KiNativ. Kinobeads are used to reversibly immobilize broad-spectrum kinase inhibitors as probes. The test compound and kinobeads compete to bind target kinases, inducing a decrease in the target associated with the kinobeads, which is identified through LC-MS/MS analysis as the protein in abundance (Fig. 10, right). Klaeger et al. presented large datasets showing the potency of 243 clinical kinase inhibitors based on this strategy and developed a novel selectivity metric termed CATDS (concentration- and target-dependent selectivity) for evaluating the selectivity of kinase inhibitors. Among the tested compounds, lapatinib, capmatinib, and rabusertib showed the highest selectivity and were applied as selective probes.^102^ The data revealed new targets for kinase inhibitors in the clinic, particularly non-kinase targets, which may explain the mode of action of these drugs under physiological conditions. For example, the bone osteosarcoma cell line U2OS is sensitive to the SRC/ABL dual inhibitor saracatinib even when the target gene *SRC* is knocked down. The researchers found that BMPRIA, which participates in bone morphogenesis, was a novel target of saracatinib and might mediate the inhibitory effect of saracatinib in U2OS cells. In addition, these results suggest the prospect of using druggable kinases, such as SIK2, in immune and cancer therapy. Combining the data on the phosphorylated proteome led to further refinement of novel pathways, determination of drug response markers, and enhancements to the theoretical basis of combination therapy.^102^ Through proper modifications, different kinobeads can be applied to profile potential covalently bound off-target irreversible kinase inhibitors.^103^ Dittus and co-workers reported a unique kinobead profiling strategy to identify new targets for irreversible kinase inhibitors.^29^ Using this approach, ibrutinib was found to bind covalently not only to TEC family kinases but also to BLK and ZAK. However, there are several limitations of chemoproteomic methods.^104^ First, kinases that do not interact with probes cannot be detected. Second, the kinase expression level of the selected cell line or tissue may be too low to be detected. Optimization and combination of the probes or complementary cell lysates can be used to extend the target coverage.^97,98,104^ The utility of the proteomic analysis method is also limited by low throughput and high cost.

Compared to proteomic analysis, bioluminescence-based analysis is more suitable for higher throughput profiling. NanoBRET is a novel profiling assay that enables investigation into compound selectivity in live cells.^105^ In this profiling, bioluminescence resonance energy is transferred between target kinases fused to NanoLuc luciferase and a cell-permeable fluorescent tracer that binds to the target kinase.^105,106^ The binding affinity of the test compound is determined on the basis of its ability to compete with the tracer and thus influence the production of BRET. Recently, NanoBRET profiling of clinical kinase inhibitors was performed with 178 kinases.^107^ By comparing the results of NanoBRET profiling with biochemical profiling data, crizotinib exhibited improved target selectivity under cellular ATP conditions, and certain off-target proteins were disengaged in live cells at the clinically relevant dose.^107^ These results indicate that profiling data in live cells might be more accurate than that of in vitro protein level profiling due to the high ATP concentration in cells. In addition, a NanoBRET panel against all 21 human CDKs was established to quantify target occupancy in live cells. The profiling results revealed a comprehensive landscape of the isozyme potency and selectivity of 46 CDKs inhibitors that have entered clinical trials. Among these compounds, BAY-1251152 exhibited the strongest target affinity and selectivity of CDK9, and it was found to be suitable for cellular research as a molecule tool. Most CDK inhibitors display affinities of more than two members of CDKs. For example, BMS-265246, which targets CDK1/2, was found to also be a potent CDK8/19 inhibitor.^108^ Although probes have been widely used in kinase profiling, the development of chemical probe-free profiling methods might have advantages for expanding the target range, particularly non-kinase targets.

### Probe-free thermal-based techniques

**Fig. 11:**
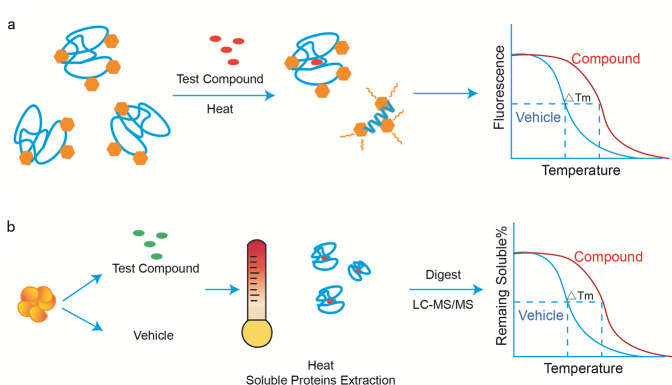
Thermal-based techniques applicated in kinase inhibitor profiling. **a** Differential scanning fluorimetry (DSF) estimates the thermal shift of target protein by fluorescence dye. The dye provides a fluorescence signal when protein unfolds. The dye is quenched when proteins fold natively. **b** Thermal proteome profiling (TPP) evaluated the change of protein *T*_m_ by cellular TSA (CETSA); the soluble native proteins are quantitated by mass spectrometry analysis

Thermal-shift assays (TSAs) determine a shift in protein melting temperatures (*T*_m_). Upon ligand binding, the proteins are thermally stabilized, which induces a shift in the protein melting temperatures.^109^ This provides the theoretical basis for the development of thermal profiling strategies such as differential scanning fluorimetry (DSF) and thermal proteome profiling (TPP) (Fig. 11).^110^

DSF detects the *T*_m_ shift of proteins by labeling purified proteins with fluorescent dyes (Fig. 11a).^110^ The dyes are quenched when proteins fold natively. When proteins unfold, the hydrophobic cores are exposed and interact with the dyes which provided a fluorescence signal. The melting curve of proteins was evaluated by the fluorescence intensity as a function of temperature.^111^ It is easy to perform on RT-PCR instruments, which is suitable for high-throughput screening.^111,112^ It has been applied in lead compounds discovery and fragment-based ligand design.^113,114^ By DSF screening against the small-molecule library, p90 RSK inhibitor BI-D1870 was found to induce a significant *T*_m_ shift of vaccina-related protein kinase 1 (VRK1).^115^ Based on this scaffold, a novel VRK1 inhibitor was developed, which showed better potency and selectivity.^116^ However, using extrinsic dyes in the DSF experiment, the signal-to-noise ratio is often limited by the protein purity and homogeneity, and the high background of the signal often leads to false-positives hits. In addition, this system is not suitable for membrane proteins in buffers containing detergent.^117^ To solve these limitations, intrinsic fluorescence was utilized in DSF. Using GFP (green fluorescent protein) fused protein in DSF, the stability of protein can be detected through the green fluorescence.^118^ Recently, nanoDSF technique was developed, which detects the intrinsic tryptophan fluorescence of proteins at 330 and 350nm^119^ with specially designed monitoring instrument Prometheus NT.48 that is commercially available (NanoTemper Technologies, Munich).

Mikhail and co-workers developed TPP for tracking cancer drugs in living cells (Fig. 11b).^120^ By combining the cellular TSA with quantitative mass spectrometry analysis, protein denaturation curves and *T*_m_ changes, which reflect the thermal stability in cells, were evaluated.^120–122^ Through subsequent comparison of the changes in protein *T*_m_ between vehicle- and drug-treated samples, the approach provides an unbiased evaluation of drug target occupancy for various targets, not just for kinases, facilitating the identification of drug efficacy and toxicity markers.^120,121^Using this strategy, Steffan and co-workers found that brusatol, which has KEAP1/Nrf2 inhibition activity, inhibited global protein synthesis.^123^ The heme biosynthesis enzyme FECH was reported to be an off-target of vemurafenib and alectinib, which explains why these inhibitors cause phototoxicity as a side effect in the clinic.^120^ In addition, this thermal profiling strategy can be used to detect potential downstream effectors of target proteins. For example, by profiling dasatinib in intact K562 cells with an active signaling pathway and in cell extracts, Mikhail and co-workers found a thermoshift of CRK/CRKL downstream of BCR-ABL. However, the direct target of dasatinib, BCR-ABL is not stabilized by its binding.^120^ Therefore, the fact that some proteins are not stabilized by ligand binding might limit the application of this assay. Besides, this assay is not suitable for membrane proteins that are not solubilized in detergent-free cell extraction buffer, low abundant proteins, and proteins with extreme melting temperature.^124,125^ Recently, the detection limit for membrane proteins has been overcome by new methods such as the application of mild detergent and enrichment of cell surface proteins.^125,126^ Furthermore, the development of mass spectrometry improved the throughput and sensitivity of this assay.^127,128^ Although this profiling is expensive and not commercially available, it might have more applications through methodological optimization, allowing the study of the drug–protein interaction in live cells, even in tissue or organs in the future.^129,130^

In summary, we can see that all of the aforementioned profiles truly detected the binding affinity of compounds with kinases in competitive or uncompetitive ways. However, in some cases, strong binding affinity does not necessarily correlate with potent inhibition of activity.^131^

### Catalytic activity-based profiling approaches

Measurement of kinase catalytic activity can be used to verify the results of a binding assay.^131,132^ The ADP-Glo™ assay from Promega is a universal assay with accuracy that has been validated with a large kinase panel spanning the kinome, and it detects the generation of ADP catalyzed by kinase.^133^ It is suitable to use not only for hit compound screening against one target but also for profiling inhibitors against large or small kinase panels using a single platform. In addition, based on time-resolved fluorescence resonance energy transfer technology, different platforms for binding affinity or kinase activity measurement have been developed that can be applied to address high-throughput screening, hit confirmation, and lead optimization programs. However, catalytic activity tested in vitro may reflect interference by many factors. For example, the lack of cofactors and the presence of truncated, fused, or tagged recombinant proteins do not always reflect the physiological kinase form.^134^ ATP concentration and the peptides used to mimic protein substrates are all factors that will affect the in vitro activity.^94^

**Fig. 12:**
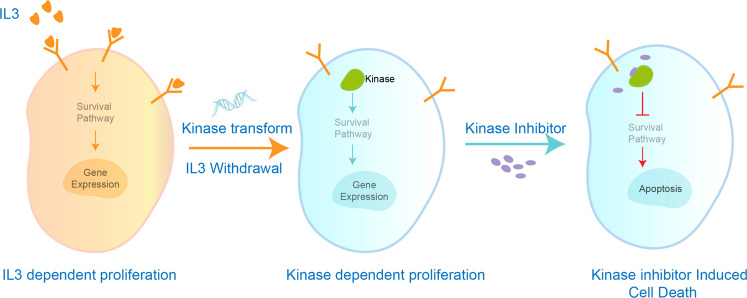
Engineered Ba/F3 kinase cells for kinase inhibitor selectivity and activity profiling. Ba/F3 cells are rendered IL-3-independent and kinase-dependent by introducing a kinase gene in Ba/F3 cells through a genetic engineering approach. The activity of the kinase inhibitor can be evaluated by cell viability of kinase-Ba/F3 cells after inhibitor treatment

A kinase-dependent Ba/F3 panel provided a strategy to evaluate kinase catalytic activity in vivo. Notably, this phenotype profile characterizes the activity through cell viability. Ba/F3 is a mouse-derived hematopoietic cell line that depends on interleukin-3 (IL-3) for proliferation and survival (Fig. 12).^135^ Expression of kinase proteins in Ba/F3 cells through a genetic engineering approach activated the kinase-dependent proliferation signaling pathway and rendered the cells IL-3-independent.^136^ In this way, cell viability was associated with kinase enzymatic activity. Many mutations of oncogenic kinases have been found to be driving forces of cancers and other diseases. By constructing kinase mutation-dependent Ba/F3 cell lines, selectivity and activity profiling between kinases and their mutants is possible, which has been particularly useful for characterizing certain fusion mutations that are not easy to express in vitro.^136^ Screening randomly mutated kinase-Ba/F3 cells by kinase inhibitors allowed one to identify site mutations that induced drug resistance.^135,137^ Using this strategy, Cools et al. performed an in vitro screening of FLT3 randomly mutated Ba/F3 cells with FLT3 inhibitors and identified that the FLT3 G697R mutation in the ATP-binding region induced resistance to FLT3 inhibitors such as PKC412.^138^ This site mutation was later found in patients treated with PKC412, indicating the validity of the in vitro predictions.^139^ However, for kinases that are not functionally related to the cell growth signaling pathway and cannot maintain the survival of Ba/F3 kinase cells in the absence of IL-3, this approach is unlikely to work.

Caliper off-chip mobility-shift assay (MSA) is most frequently used for drug discovery such as small-molecule screening, fragment-based screening, target specificity (profiling), and mechanism of action studies. This assay can be applied to a wide range of proteins, including kinases, phosphatases, lipid kinases, proteases, phosphodiesterases, nucleic acid-binding proteins, and epigenetic targets (HATs, HDACs, and methyltransferases). Caliper off-chip MSA technology uses capillary electrophoresis in a microfluidic environment to analyze enzymatic assays. The Caliper LabChip platform is based on microfluidic chips containing a network of miniaturized, microfabricated channels through which fluids and chemicals are moved to perform experiments. By applying an electric potential difference across the separation channel, fluorescently labeled substrates and products are separated by electrophoresis and detected by light-emitting diode-induced fluorescence. Both the substrate and the formed product were detected and measured for each sample. The amount of product formed is determined by calculating the ratio of product peak heights. For kinetic analysis, the reaction is monitored as it progresses by sequentially “sipping” samples onto the chip at various time intervals.^140,141^ The kinase activities of many kinase inhibitors have been evaluated by caliper off-chip incubation MSAs. The major advantages of this method are high sensitivity and no use of termination or quenching reagent, but the assay usually takes a long time for detection.

## Advances in efficacy assessment models and technology

**Fig. 13:**
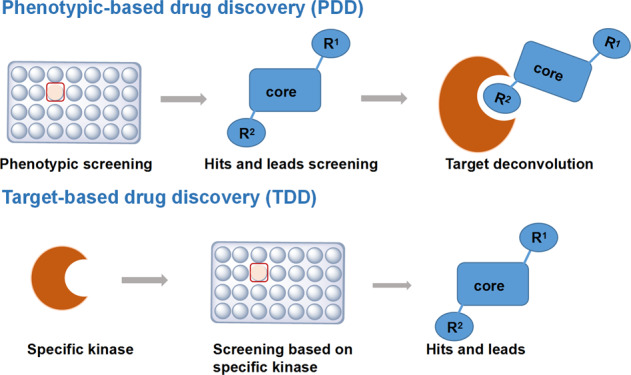
The drug discovery and development process. Phenotypic-based drug discovery (PDD) approaches are measured through a physiologically relevant system such as animal or primary cells. The molecular mechanism of action is unknown previously; target-based drug discovery (TDD) features anchoring to a defined target kinase or protein for screening a fit compound

Drug discovery is an expensive, low efficiency, and highly risky business. An analysis suggests that it costs more than $1 billion to successfully develop a small-molecule kinase inhibitor, and takes 12.5 years on average. Reliable assessment efficacy models and drug screen technologies can accelerate the discovery and development of kinase inhibitors. Target-based drug discovery (TDD), also known as reverse pharmacology, which features anchoring to a defined target kinase or protein, has long been favored by pharmacologists due to its rational basis for drug design. Compared to phenotypic screening, the TDD approach is more target-specific and requires known targets, which are sometimes very difficult to identify. In addition, TDD makes SAR exploration easier and faster, hence offering a better chance to obtain the “best-in-class” inhibitors (Table 2). In recent years, phenotypic-based drug discovery approaches become more and more popular. With this approach, the properties of compounds are measured through a physiologically relevant system such as animal or primary cells, the previous understanding of the molecular mechanism of action is unnecessary, and drug identification follows an unbiased approach (Fig. 13). A well-known example of a drug discovered by phenotypic screening is aspirin, whose mechanism of action was identified after approximately 100 years. The majority of first-in-class drugs identified from 1999 to 2008 were the culmination of phenotypic drug discovery approaches based on either in vitro or in vivo screening models that mimicked the disease state of interest.^142,143^ Rapamycin, an FDA-approved first-in-class drug, was identified through phenotypic screening in a fungus model and later was developed as an antifungal drug on the basis of its potent immunosuppressant activity, which was identified through phenotypic screening in vivo.^144^ In 1991, the true mechanistic target of rapamycin immunosuppressant activity was discovered, and the target was mTOR.^145^ Some unusual kinase activators were also found in this way; e.g., MLR-1023, a potent and selective allosteric LYN kinase activator, was discovered through an in vivo phenotypic screening platform, and MLR-1023 has been used alone or in combination with metformin to treat type 2 diabetes in clinical trials (NCT02317796 and NCT03279263).^146^ Some kinase inhibitor hits were discovered soon after this approach was implemented; for example, the CDK9 kinase inhibitor MC-180295 was found after the optimization of SNS-032, which had been identified through a phenotypic screening approach.^147^ Moreover, dual TTK protein kinase/CDC2-like kinase (CLK2) was discovered through in vivo phenotypic screening of a triple-negative breast cancer tumor cell line.^148^ Novartis reported that the multitarget compound GNF3809-protected rat insulinoma β cells from death induced by proinflammatory factors, which was also discovered by phenotypic screening in vitro.^149^

**Table 2:** A comparison of target-based and phenotypic approaches

	Target-based drug discovery (TDD)	Phenotypic drug discovery (PDD)
Target	Known	Unknown
Cost	Low	High
Speed	Rapid	Moderate
Ease of SAR	Easy	Difficult
Characteristics of drug	Best-in-class	First-in-class
Clinical translatability	Lower (depends on other evidence)	Presumed to be higher in general

### Patient-derived tumor cells

In addition to canonical in vivo animal- or in vitro cell line-based phenotypic screening, patient-derived tumor cells (PDCs), derived through a short-term culture of primary cells collected from patients, have recently attracted considerable interest for use in novel phenotypic screening and personalized cancer therapy.^150^ PDCs are thought to be useful surrogates of patient tumors in the clinic because of their obvious advantages over other preclinical models. First, PDC models faithfully retain the histological and genomic features of primary patient tumors. Second, the labor and time needed to build the PDC models are reduced. Normally, PDC models can be obtained within 2 and 3 weeks, making them more feasible for use in clinical application than other methods. Third, the success rate of model establishment is as high as 73.6% in patients with metastatic cancer. Fourth, PDC models are applicable to high-throughput drug screening. Finally, the concordance between clinical response and drug susceptibility is high.^150^ We and others have shown that low-passage patient-derived primary cells established from acute myeloid leukemia patients, chronic myeloid leukemia (CML) patients, gastrointestinal stromal tumor (GIST) patients, and sarcoma patients can be used for screening and identifying novel kinase inhibitors in preclinical studies.^151,152^ Lee et al. tested the therapeutic landscape of 60 molecule-targeted compounds on 462 PDCs across 14 cancer types and identified lineage-specific drug associations, such as gastric cancers and PI3K inhibitors.^153^ Brodin et al. evaluated the sensitivity of 525 anticancer agents on patient-derived sarcoma cells and found that the drug sensitivity of the patient sarcoma cells in vitro correlated with the response of the patient to the treatment received. They proposed that dasatinib, a c-Src inhibitor that they identified through this research, is an effective drug against sarcomas carrying chromosomal translocation.^154^

### Organoid/3D cultures

The first patient-derived tumor organoid culture was established in 2009, and since then, the explosive growth of organoids as a novel ex vivo model has revolutionized preclinical cancer research over the past decade.^155^ Organoids are thought to be superior models for identifying and testing novel anticancer drugs due to their unique characteristics; for example, (i) organoids stably retain the genetic and phenotypical features of the parental primary tumors; (ii) organoids from both healthy and tumor tissues can be generated, which allows screening of drugs that specifically target tumor cells while leaving healthy cells unharmed; (iii) organoids can be expanded long term and be cryopreserved, allowing for the generation of living tumor organoid biobanks; (iv) organoids can be generated from 4 to 6 weeks, which is a major advantage for patients with advanced stages of cancer; and (v) organoids are suitable for medium-to high-throughput drug screens.^155–157^

Organoids have been successfully generated from primary tumors of the breast, colon, pancreas, lung, head and neck, ovary, and prostate.^155,156,158,159^ In fact, there have been some promising results in terms of novel targeted molecular drugs identified with organoid models. Doublecortin-like kinase 1 (DCLK1) is a serine/threonine kinase that is upregulated in a wide range of cancers, including colorectal cancer and gastric cancer. DCLK1 expression is particularly relevant in pancreatic ductal adenocarcinoma (PDAC), where its high expression correlates negatively with PDAC patients’ lifespan.^160^ Ferguson et al. worked on the development and characterization of DCLK1-IN-1 inhibitors. They used organoid samples isolated from DCLK1-expressing patients as preclinical testing models and found that DCLK1-IN-1 is a potent and highly specific inhibitor of DCLK1 and DCLK2 kinases.^161^

### Patient-derived xenograft

The prevalence of patient-derived xenograft (PDX) models was established in the early 2000s because of significant limitations in cell line-based models that were commonly used in the discovery of novel drugs in clinical trials. PDX models are generated by the removal of primary or metastatic tumor specimens from patients and followed by surgical engraftment into immunocompromised mice; these models have emerged as a useful model for cancer research and have been widely used in novel anticancer agent screening.^162^ In the PDX model, both the genetic and pathohistological characteristics of tumors are preserved. Tumor heterogeneity was shown to be similar to that of the parental patient tumors. Furthermore, the tumor architecture and tumor microenvironment closely resemble those of the original tumor tissues. All of these characteristics suggest that PDXs are useful preclinical models for mechanistic research on tumor progression and for predicting the therapeutic effects of novel drug candidates.^163^ To date, multiple PDX models have been established from a wide range of human cancer types, including lung cancer, ovarian cancer, breast cancer, pancreatic cancers, gastric cancer, and CRC, and they have been used in preclinical research.^155,164^ In addition, PDX models have also been widely used for the development of novel molecular targeted drugs and evaluation of the drug sensitivity patterns of primary tumors. Using PDX models, Wang et al. investigated the antitumor activity of pyrotinib, an irreversible pan-HER TKI, and found that pyrotinib shows potent antiproliferative activity against non-small cell lung cancer with HER2 exon 20 mutations. In a clinical trial, pyrotinib also showed promising efficacy, which validated the PDX findings obtained in preclinical testing.^165^ BTK is a key mediator of BCR-dependent cell growth signaling and a promising therapeutic target for multiple diseases, particularly in hematopoietic malignancies such as MCL. Li et al. investigated the molecular mechanisms of BTK inhibition with the novel molecular targeted inhibitor BGB-3111 (zanubrutinib) in MCL models.^166^ They found that BGB-3111 exhibits greater specificity for BTK, and it exhibits excellent efficacy in preclinical MCL cell models and PDX models, which will be helpful in promoting the progress of clinical trials. In addition, PDX models have also been used to evaluate the effect of different molecular targeted drugs against different cancer types, including GISTs, melanoma with BRAF(V600) mutations, and CRC with KRAS G12C mutation.^167^

### DNA-encoded chemical library

**Fig. 14:**
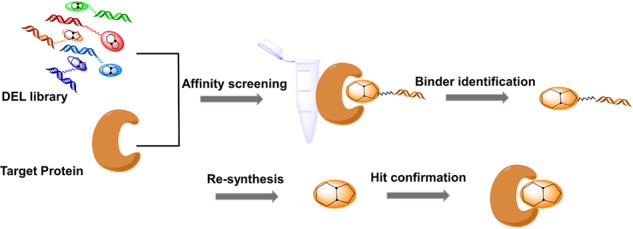
Schematic diagram of drug screening using a DNA-encoded chemical library (DEL). DEL is a new emerging screen platform for seeking bioactive compounds from millions to billions of small molecules with unique DNA sequence tags attached

A DNA-encoded chemical library (DEL) is a new screen platform seeking bioactive compounds from millions to billions of small molecules with unique DNA sequence tags attached, which is based on the binding affinity screening of pharmaceutically relevant proteins (Fig. 14). This capability allows compound screening and hit selection to be rapidly performed. With more chemical diversity than other methods, the use of a DEL is a highly efficient approach to explore larger chemical spaces for drugs. DELs have been widely adopted and employed in numerous drug discovery programs.^168^ GSK reported the identification of two compounds, the sEH (soluble epoxide hydrolase) inhibitor GSK2256294 and RIPK1 inhibitor GSK2982772 using the DEL hit-finder approach, and both of these inhibitors are currently being tested in clinical trials.^169,170^ Although billions of compounds can be screened in a small tube, the limitations of the technology are that compounds should be soluble and target proteins either tagged or immobilized on a solid support should be purified. Moreover, only a few kinds of reactions have been reported for the construction of DEL.^171^

### Artificial intelligence

**Fig. 15:**
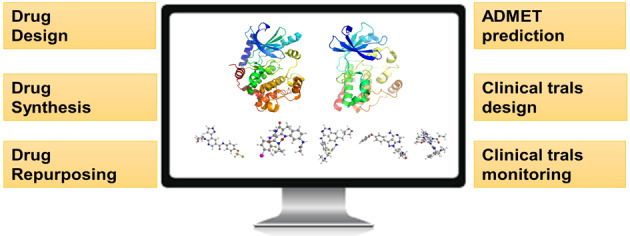
Artificial intelligence (AI) in kinase inhibitor discovery. AI is integrated in various technologies in a network system that can mimic human intelligence. AI is widely used in drug discovery and development, which is applied in the drug discovery and development to improve pharmaceutical products including design, synthesis, repurposing, ADMET prediction, and even the clinical trials design and monitoring

It is estimated that there are approximately 10^60^ compounds in the chemical space, and only a tiny proportion of these molecules have been developed as drugs. Artificial intelligence (AI) is integrated various technologies in a network system that can mimic human intelligence. AI is widely used in drug discovery and development, which is applied in drug discovery and development to improve pharmaceutical products including design, synthesis, repurposing, ADMET prediction, even the clinical trials design, and monitoring. We mainly focus on the application of drug design and repurposing (Fig. 15). Numerous efforts have been made to modify the drug map for a long time, which causes continual increases in the research and development costs of developing a new drug. A computer-assisted drug design (CADD) strategy has been widely used to guide the exploration of the drug space; however, the biological activity of compounds can seldom be predicted precisely. As no formula can be established to precisely describe the interactions between molecules and targets, automating drug research and development through machine learning from a large number of samples represents a new option.^172,173^ Traditional computational methods rely on manually programmed logical operations to process information and optimize tasks that are difficult or time-consuming for humans to understand or complete, such as performing a linear regression on a set of data. AI, on the contrary, can solve nontrivial tasks on the basis of human-defined rules.

Many kinase inhibitors with enhanced predicted activities and binding affinities, which can serve as lead compounds for further synthesis and preclinical testing, have been designed through in silico modification. Many new chemical scaffold kinase inhibitors (imidazopyridazine, imidazopyridine, isoquinoline, phenazinamine, etc.) have also been designed by computational approaches and represent good examples of the discovery of new kinase inhibitors.^174^ ETC-206, an oral MNK 1/2 kinase, was designed from a previous compound through homology modeling, docking, molecular dynamics (MD) simulations, and free energy calculations, and it was investigated for synergism with dasatinib in vivo and is currently in a phase I clinical trial with patients with blast crisis CML.^175^ CADD techniques (molecular docking, similarity, and pharmacophore searches) were used in the design of the allosteric ABL inhibitor asciminib, which is currently in clinical trials.^176^

Computational approaches, such as quantitative SAR (QSAR) modeling, ligand- and structure-based virtual screening, molecular docking, MD, quantum mechanics, fragment-based drug design, and machine deep-learning methods, all provide unique insight into the conformational landscape of kinases, structural requirements for inhibitory activity, binding modes, and atomistic mechanisms of allostery, which represent indispensable information for rational de novo design.^177^ The goal of QSAR modeling in drug discovery is to find a mathematical relationship that connects molecular properties (as encoded in molecular descriptors) with some quantitative activity metric of a compound. QSAR models have become important tools in AI-aided drug design, such as virtual screening and lead optimization. Jiang and co-workers provided new chemical starting points for further structural optimization of FGFR1 inhibitors by combining pharmacophore-based 3D-QSAR analysis and virtual screening approaches.^178^ Recently, an increasing number of people have developed new QSAR models and applied them to the discovery of kinase inhibitors. Koneru et al. integrated the fragment-based drug discovery technique with QSAR and MD to design the second-generation SRC kinase inhibitor RL-45.^179^ QSAR has also been used in LBVS to predict the properties of new chemical compounds. Several potential kinase inhibitors, such as a dual ALX/FLT3 inhibitor and a CDK9 inhibitor, were reported on the basis of LBVS methodology.^180,181^ There are many cases of QSAR models used in the design of kinase inhibitors for VEGFR2,^182^ EGFR,^183^ MCL-1,^184^ IKK-β,^185,186^ LYN,^187^ and MER,^188^ and so on.^177,178^

Based on QSAR, virtual screening, and deep-learning models, AI-aided de novo drug design became possible. Compared to traditional wet lab-based drug discovery, AI-assisted de novo drug discovery has the advantage of time and cost efficiency as long as the deep-learning techniques used are well developed and basic learning data provided are sufficient. Based on the calculated GScore, the fragment-based drug design method has been used to create new BCR-ABL inhibitors. De novo designed molecules have better inhibitor capacity than the most commonly used tyrosine kinase inhibitors on the market. These molecules have shown strong potential to become drugs capable of inhibiting all mutations, particularly the BCR-ABL-T315I mutant, which is now the leading cause of death due to the difficulty of controlling its inhibitors.^189^ As an example of a proof-of-concept for developing drug via AI, a preclinical lead compound of DDR1, a kinase target implicated in fibrosis and other diseases, was recently discovered in an AI-assisted de novo drug design approach within 21 days. This approach uses a new deep-learning technique called generative tensorial reinforcement learning, through which synthetic feasibility, novelty, and biological activity can be optimized.^190^

### Silicon-based kinase profiling methods

Recent advances in deep-learning algorithms have made the profiling of small molecules possible. Many in silico modeling approaches have been developed to predict kinase inhibitory activity for large-scale compound libraries. Three online platforms are currently available for use in predicting kinome-wide targets: KinomeX, KinomeFEATURE, and ProfKin. KinomeX is based on an established multitask deep neural network model trained with over 140,000 bioactivity data points for 391 kinases to predict the kinome-wide poly-pharmacology effect of small molecules for designing novel chemical modulators and drug repositioning.^191^ Extensive computational and experimental validation has been performed using the KinomeFEATURE database to predict the off-targets of small molecules, which is achieved by searching for kinase-binding sites through comparisons of protein structural microenvironments.^192^ The KinomeFEATURE database and other auxiliary information used for performing kinase pocket similarity analyses can be downloaded from the Stanford Sim TK website (https://simtk.org/projects/kdb). Based on a KinLigDB (kinase-ligand-focused database), ProfKin, which provides the complex structures of 4219 kinase ligands covering 297 human kinases, associated information, binding site type, ligand binding type, interaction fingerprints, downstream signal pathways, and related human diseases, has been exploited as a multifunctional website for structure-based kinase selectivity profiling.^193^ The website (http://www.lilab-ecust.cn/profkin/) can be used to predict the possible binding modes, which can be helpful for target prediction and mechanistic study.

## Challenges and future perspectives

New strategies, technologies, and methods undoubtedly have expanded the kinase inhibitor discovery platform and accelerated the discovery process, increasing the number of diversified preclinical candidates for clinical development. All of these new approaches have been tried in efforts to overcome a specific hurdle or solve an identified problem, such as challenges to time efficiency, cost efficiency, and even knowledge efficacy during the discovery journey, but inevitably, these new methods exhibit their limitations. Because of the evolutionary pressure within cancer cells, drug resistance seems an endless problem. Therefore, to take full advantage of these new approaches, combinations of new approaches and traditional approaches might make these strategies more powerful. Among the approved small-molecule kinase inhibitors, small molecules targeting kinases of DNA-damage response signal pathways such as ATR, ATM, DNA-PK, and CHK1 are still in clinical trial stages; more molecules can be designed of these kinases. Recently, a novel selective ATR kinase inhibitor shows potent antiproliferative activity against a broad spectrum of human tumor cell lines and exhibits strong monotherapy efficacy in cancer xenograft models;^194^ its antitumor responses were also observed in patients with advanced solid tumors.^195^ With the quick development of cancer immunotherapy, many kinases involved in tumor immunology such as CSF1R,^196^ HPK1,^197^ etc. can be targeted by small molecules. There is still a lot of room for the development of protein degradation technology, e.g., the PKs of PROTACs. In addition, other degradation technology such as lysosome-targeting chimeras for degradation of extracellular proteins,^198^ merging PROTAC, and molecular glue for degrading may become new directions.^199^ Research showed that many disease pathology is driven by protein ubiquitination and degradation; thus, DUBTAC (de-ubiquitinase-targeting chimera) platform for targeted protein stabilization of specific proteins was developed, which is contrary to PROTACs.^200^ The hetero-bivalent system is a new class of molecules that possess three components including an ATP-competitive ligand and pseudo-substrate peptide that are covalently connected through a linker. This unique rational design permits the simultaneous binding to both ATP and peptide binding sites and thus making these compounds possess high selectivity and activity, which can meet the needs of the development of more potent and selective kinase inhibitors.^201^ In addition, with the development of technology several new promising protein–protein interaction compounds (PPIs) have entered clinical studies. PPIs modulators can be a new strategy to design small molecules and peptide inhibitors targeting specific kinases domains.^202,203^ Many kinases such as NLRP3, JNK perform their functions by forming complexes with their interacting partners or substrates, and the interactions between them are also involved in the regulation of kinase functions, which provides new opportunities for designing PPI inhibitors that work through blocking these interactions.

In addition to small molecules alone, combinations with large molecules, such as conjugated antibody-drug compounds, may unleash the full potential of small-molecule kinase inhibitors. Despite the variety of innovations applied to kinase inhibitor discovery, from screening, design, and in vitro and in vivo evaluation, the rate of successful development of drugs that can be approved for delivery in the clinic has not significantly improved. One reason for the ultimate failure of most drugs in clinical trials is the result of most innovations being focused on the early discovery stage. Clearing the many hurdles in the preclinical development stage of kinase downregulators also requires extensive empirical knowledge. With more new technologies starting to impact these efforts, such as the use of AI for the prediction of physicochemical and ADMET properties, we believe that more diverse kinase inhibitors will be delivered to the clinic to fulfill the heterogeneous clinical demands in the near future.
